# Advantages evaluation of integrating administrative and functional zones based on Island political location potential theory: A case study of the Zhoushan Archipelago New Area in China

**DOI:** 10.1371/journal.pone.0320687

**Published:** 2025-04-01

**Authors:** Linhan Shao, Tingting Xu, Kaiyong Wang, Yufan Chen

**Affiliations:** 1 Ningbo University Donghai Academy, Zhejiang Ocean Development Think Tank Alliance, Ningbo, Zhejiang, China; 2 Department of Geography and Spatial Information Techniques, Zhejiang Collaborative Innovation Center & Ningbo Universities Collaborative Innovation Center for Land and Marine Spatial Utilization and Governance Research, Ningbo University, Ningbo, Zhejiang, China; 3 Key Laboratory of Regional Sustainable Development Modeling, Institute of Geographic Sciences and Natural Resources Research, Chinese Academy of Sciences, Beijing, China; Czestochowa University of Technology: Politechnika Czestochowska, POLAND

## Abstract

Administrative division adjustment is a new reform method and policy tool designed to align with local economic foundations and revitalize the development potential of administrative districts. However, it lacks systematic and quantifiable theories and methods. In response, we propose the Political Location Potential (PLP) model based on production functions, which is specifically applicable to islands. Using the Zhoushan Archipelago New Area as a case study, we analyze the efficiency and mechanisms behind the establishment of China’s first archipelago new area. The PLP model consists of two key components: horizontal production resource allocation capability and vertical administrative management authority. The PLP of Zhoushan City has increased from 0.007 in 2008 to 0.066 in 2022, with a relative potential energy of 2.853, 3.717 and 1.449 during the critical stages of 2010–2014, 2008–2013 and 2013–2018, respectively. Competitive comparative advantage and land-island connectivity capability have significant direct and indirect effects on the PLP of the island, but the impact effect shows a lag effect, with a lag period of 3–5 years. The theory and model of the PLP offer strong explanatory power for the effects of integrating administrative and functional zones, providing a fresh analytical perspective for local administrative division planning.

## Introduction

Administrative districts are fundamental to a nation’s political structure, serving as essential tools for central government oversight and governance [1,2]. Administrative district adjustment (ADA) plays a key role in achieving efficient administrative boundaries and meeting socio-economic development needs [3,4]. Compared to other administrative entities, districts hold a unique position due to their location, hierarchical level, authority, and jurisdiction. The concept of political location potential (PLP) refers to the status and latent capacity of a district within its administrative framework, comprising two components: potential scope (the horizontal variation in jurisdiction) and potential energy (the vertical shift in administrative hierarchy) [5,6].

Changes in administrative boundaries can alter a region’s political standing, affecting its market scope and the local government’s administrative and fiscal authority, which in turn influences economic growth and regional development [7]. Functional zones are quasi-administrative districts with some management responsibilities but lacking full administrative authority [8]. The creation of state-level new districts, such as the Zhoushan Archipelago New Area in 2011, exemplifies the integration of administrative and functional zones, granting elevated economic management authority and affecting the PLP. The fusion of administrative districts and functional zones combines oversight with specialized management, optimizing resource allocation, improving services, and promoting balanced economic growth, particularly in rapidly developing areas like islands or coastal regions [2,9].

While some studies have examined the evolution of administrative divisions, the impacts of the ADA, and the transformation and development of functional zones, there remains a significant gap in systematic theoretical and quantitative analysis, particularly from the perspectives of geographical and institutional space. This is especially true when considering cases where the administrative level remains unchanged but the economic authority of the region is upgraded. The dynamic relationship between administrative boundaries, institutional reforms, and economic authority has not been fully explored, and the complex spatial and institutional shifts occurring in such adjustments warrant a more comprehensive theoretical framework. This gap calls for an integrated approach that combines geography, institutional theory, and economic analysis to better understand the multifaceted nature of such changes and their long-term implications for regional governance and development. While most studies focus on the economic development of new districts through industry-city integration, and rely mostly on qualitative analysis and basic quantitative methods like Differences-in-Differences. This study focuses on the Zhoushan Archipelago New Area, trying to ask three questions:

(1) How do the ADAs impact the economic development of new districts, particularly in terms of supporting grassroots governance in China?
(2) What are the positive and negative externalities associated with the establishment of state-level new areas through the lens of administrative district institutional space?
(3) How can a PLP model be applied to island areas to identify key factors influencing PLP and assess the impact of state-level new areas on island development?

## Literature review

Administrative districts are the core units for hierarchical management and play a vital role in state governance. As China’s social economy and urbanization progress rapidly, these districts evolve to serve different roles at various stages. Unlike China’s administrative system, Western countries often have independent central urban areas and suburban towns, each functioning as separate political entities [10]. Integrating administrative and functional areas involves merging economic function zones with administrative districts, where the government not only maintains the economic functions but also assumes political and social responsibilities. The creation of state-level new areas is closely linked to such integration, influencing regional coordination and macro-strategic planning. These new areas, with broader strategic positioning, economic functions, and institutional innovation, surpass previous regional policies such as development zones and high-tech zones [11,12].

Functional areas, which are special administrative districts, have attracted attention both domestically and internationally. Chinese scholars analyzed the spatial relationship between administrative and functional districts, focusing on their role in resource allocation [13]. Gu et al. developed a framework for studying the ADA in functional areas, particularly for large cities or urban agglomerations [14]. Internationally, scholars such as Brush used “flow” maps to illustrate regional connections [15], and Platt introduced the concept of Areal Functional Organization, emphasizing horizontal regional interconnections [16]. Additionally, functional zones, especially development zones, are more susceptible to the impact of public crises due to their special economic functions. Major public emergencies, such as public health incidents, natural disasters, accidents, and social security issues, inevitably expose the enterprises within these zones to the risk of supply chain disruptions, leading to delays, shortages, and increased operational costs [17,18]. This, in turn, negatively impacts economic efficiency, disrupts local business operations, and hampers overall regional development, making it harder for affected areas to recover quickly and return to normal economic activity.

Research on the impact of the ADAs in China focuses on three main areas: economic effects, spatial effects, and power dynamics. In terms of economic effects, Li emphasizes the importance of adjusting administrative divisions for western China’s development. Through qualitative and historical analysis, he shows how these adjustments promote cooperation between central cities and surrounding areas [19]. Liu et al. further show that dismantling towns to create cities increases autonomy and boosts industrial land sales, using micro-level land transaction data and a difference-in-differences model [20]. Regarding spatial effects, ADAs accelerate urbanization, expand central urban zones, and increase city sizes [21]. For instance, Yin et al., using spatial econometric models and panel data analysis, found that ADAs have transformed the Pearl River Delta from urbanization to metropolitan status [22]. Lastly, in terms of power dynamics, ADAs lead to resource reconfiguration and spatial reorganization, fostering a new grassroots governance model in China [23]. This is exemplified by the abolition of townships and the establishment of county-level streets, which centralize administrative power [24]. In contrast, international research on ADAs often focuses on socio-economic restructuring during de-territorialization and re-territorialization [4,9,25–27], with scholars noting that decentralization changes regional administrative and financial authority, acting as a catalyst for growth [28,29]. Additionally, Redding and Sturm use the division and reunification of East and West Germany as a case study to show how ADAs impact market size and regional development [30].

Chinese scholars have expanded research on ADAs by introducing the concept of the PLP and developing quantitative measurement models, such as the Cobb-Douglas production function, spatial field energy models, and power exponential function. For example, Chen et al., by employing the Cobb-Douglas production function and integrating indicators such as regional economic development and transportation accessibility, demonstrated a significant increase in Chongqing’s PLP after it became a municipality directly under the central government [5]. Wang et al. developed a PLP model based on indicators of administrative resource allocation and competitiveness level, using econometric modeling to reveal that ADAs can raise PLP and boost local economic performance [6]. Wang and Feng utilized panel data analysis from 2005 to 2018 and incorporated urban agglomeration degree to analyze the relationship between PLP and the administrative economy, expanding the focus from individual districts to entire urban regions [31].

Overall, while ADAs are a response to the challenges of rapid urbanization, they do not fully address the economic difficulties faced within administrative districts. To improve the effectiveness and diversity of ADAs in China, a more in-depth analysis is needed on how to better integrate administrative and functional zones. The complexity of urban factors means that the success of ADAs can vary significantly from one area to another, which highlights the need for more comprehensive evaluations and a broader range of methodologies. Additionally, there is a need for stronger and more effective applications of PLP models to fully capture the dynamics at play and improve the impact of ADAs in different urban contexts.

## Theory and methods

### Theoretical framework of the PLP

Location potential is a concept often used in physics and sociology [32,33], but in this study, it refers to the position and competitiveness of an administrative district within a country. The PLP changes with the ADAs, such as change in administrative level, jurisdiction, or subordination. It helps explain the motivations and impacts of ADAs on regional development. The PLP influences the overall strength and growth potential of an administrative district, making it a key tool in government governance.

**Fig 1:**
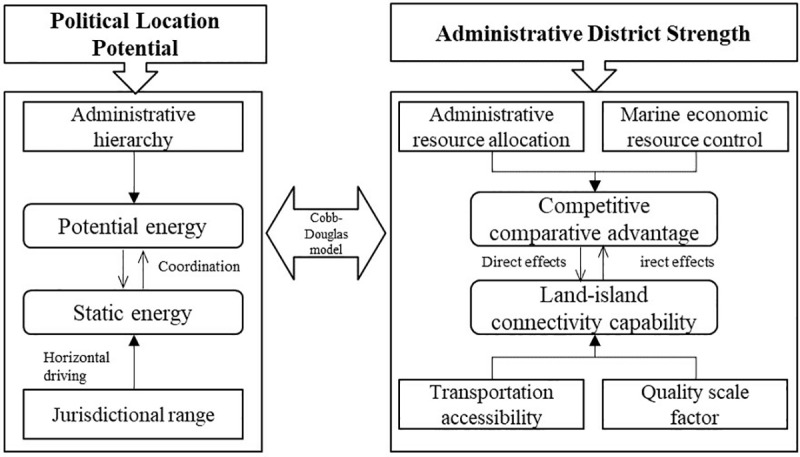
Interaction mechanism on political location potential.

When analyzing PLP, two key dimensions should be considered: horizontal jurisdictional potential and vertical administrative potential. Horizontal potential refers to the resource allocation power driven by factors like administrative level and financial authority. Vertical potential, on the other hand, reflects the district’s development capacity, shaped by its jurisdiction, population, industries, and resources. These factors together define the administrative district’s economic power and spatial combination [5,6,31]. After ADAs, such as upgrading or converting counties to cities or obtaining sub-provincial economic management authority, an island’s administrative hierarchy (vertical PLP) and jurisdictional scope (horizontal PLP) change, which affects its competitive advantage and connectivity (Fig 1).

The framework suggests that the ADAs directly alter the PLP by impacting the allocation of production factors in the market and the local resource environment. Changes in administrative, financial, and socio-cultural power influence how resources are allocated and invested by the state. Capital transfers from higher to lower levels of government, along with the competition and cooperation between governments at the same level, influence the flow and clustering of regional production factors. The efficiency of resource allocation—determined by the movement and concentration of factors like human resources and capital—plays a crucial role in driving regional development. For islands, the allocation of human resources, capital, and coordinated urban-rural development can measure their administrative capacity, while marine economic control is measured by funding levels, marine economic activities, and investment.

Additionally, ADAs indirectly affect the external connectivity and scale of development, further influencing the PLP. The island’s geography limits its transportation options, with land-island connectivity reflecting the strength of its transportation and economic links, influenced by factors like transportation modes and travel time. The population and economic scale of an administrative district show its competitive strength. We introduce two factors—quality and quantity—to measure development. Quality measures improvements in social and economic activities (e.g., per capita GDP), while quantity tracks changes in population size.

There is an interactive relationship between PLP and these factors. When administrative resources are effectively managed, marine economic resources are optimized, and transportation is efficient, the PLP improves, strengthening the district’s position within the national administrative framework. A higher PLP boosts the district’s capacity to secure administrative and development resources, which in turn positively influences transportation and socio-economic growth.

### PLP measurement based on the Cobb-Douglas production function

We use the Cobb-Douglas production function model because it effectively captures the relationship between key factors like land-island connectivity, administrative development, resource allocation, and marine economic control. Its ability to model the impact of multiple inputs on economic output makes it suitable for this analysis. The formula is as follows.


$$Y_{PLP}=\text{k}f{\left(m,l\right)}^{\text{β}}f{\left(d,q,s\right)}^{\delta }$$ (1)

where $Y_{PLP}$ represents the political location potential of the island, $f\left(m,l\right)$ captures the direct effect of competitive comparative advantage, consisting of the island’s administrative resource allocation ability and marine economic resource control level, $f\left(d,q,s\right)$ reflects the indirect effect of land-island connectivity capability, incorporating transportation accessibility and both qualitative and quantitative development factors. Here, *β* and *δ* are the elastic coefficient of each variable with respect to the growth of PLP, and *k* is the random interference item.

In this study, based on the PLP concept and the existing research, we select relevant indicators related to the integration of administrative and functional zones and construct the corresponding formula models [5,6,31]. The coefficient settings are one of the key elements in the formula. The constant *K* is generally set to 1 based on standard practice. The settings for other coefficients are determined using expert scoring methods, with reference to case studies in Shanghai and Hebei [33,34], where β=0.3 and δ=0.2.

Local development policies may be adjusted due to the ADA, mainly manifested in significant changes in the flow and aggregation of production factors such as capital investment and technical talents, resulting in changes in the efficiency of regional resource utilization [34]. We developed an evaluation index system for competitive advantage based on two dimensions: island administrative resource allocation and marine economic resource control (Table 1). Since PLP reflects the competitiveness of an administrative district relative to others, we focused on ratios between districts rather than absolute values.

**Table 1:** Indicators for competitive comparative advantage and their significance.

Indicator		Meaning
**Ability of island administrative resource allocation**	General fiscal revenue (GFR)	The capacity of local governments to secure administrative funding
	General fiscal expenditure (GFE)	The scale of financial resources independently managed by local governments
	Total investment in fixed assets (TIFA)	The level of fiscal transfers received by local governments
**Level of marine economic resource control**	Total labor productivity (TLP)	The control of human resources in the development of the marine economy
	Export value of goods (EVG)	The degree of openness of the marine economy to external influences
	Per capita disposable income (CDI)	The urban-rural interaction dynamics within the marine economy

Dimensionality was addressed through data standardization, and weights and scores were calculated using the entropy method. After processing, the weight coefficients for the six variables GFR, GFE, TIFA, TLP, EVG and CDI are 0.130, 0.153, 0.209, 0.179, 0.140 and 0.188 respectively. The calculation formula for $f\left(m,l\right)$ is as follows.


$$f\left(m,l\right)=GFR^{0.130}+GFE^{0.153}+TIFA^{0.209}+TLP^{0.179}+EVG^{0.140}+CDI^{0.188}$$ (2)

For the Land-island connectivity capability, we used the gravity model, resulting in the following formula:


$$f\left(d,q,s\right)=\frac{Q\times S}{D_{ij}}=\frac{\sqrt{GDP_{i}\times POP_{i}}\times \sqrt{GDP_{j}\times POP_{j}}}{\sum_{m=1}^{n}{\left(\lambda_{m}\times T_{ij,m}\right)}^{b}}$$ (3)

where $D_{ij}$ is the comprehensive transportation distance from district *i* to district *j*, *Q* and *S* represent qualitative and quantitative factors of the development scale, respectively, $\lambda_{m}$ is the weight of the $m_{th}$ transportation mode, $T_{ij,m}$ is the travel time for transportation mode *m*, *n* is the number of transportation modes, b is the distance friction coefficient. Scholars such as Gu et al. have pointed out that when b=1, it is more suitable for analyzing the urban network connectivity in small-scale regions [14,35]. As Ningbo-Zhoushan represents a small-scale urban agglomeration, we have continued with this value. For calculation, we used highways and shipping, assigning weights of 0.65 and 0.35, respectively, based on travel time and cost [36–40]. We then normalized the transportation capacity using linear proportional normalization to make it dimensionless for subsequent PLP calculations. Finally, we define the relative potential energy (ΔY) of the administrative district as the ratio of the PLP before and after the ADA:


$$\Delta Y=\frac{Y_{PLP,t}}{Y_{PLP,0}}=\frac{\text{k}f{\left(m,l,t\right)}^{\text{β}}f{\left(q,d,s,t\right)}^{\delta }}{\text{k}f{\left(m,l,0\right)}^{\text{β}}f{\left(q,d,s,0\right)}^{\delta }}$$ (4)

where *t* refers to the year after the ADA, and 0 refers to the base year before the ADA.

Relative potential energy ΔY>1 indicates an increase in PLP after the ADAs, meaning the current comparative advantage is greater than before. Conversely, ΔY<1 suggests a decrease in PLP, weakening the comparative advantage. If ΔY=1, the ADA has no impact on the district’s regional advantage.

### Study area and data sources

#### Overview of Zhoushan Archipelago New Area.

China is a unified, centralized nation, and its administrative districts play a key role in governance, serving as the foundation for power redistribution and policy formulation. Currently, China has 19 state-level new areas, which are crucial for regional economic policies and growth. The establishment of such areas began with the Shanghai Pudong New Area in 1992, followed by Tianjin Binhai in 2006 and Chongqing Liangjiang in 2010. In 2011, China established the Zhoushan Archipelago New Area, aligning with Zhoushan City’s administrative jurisdiction. This new area holds sub-provincial economic management authority, surpassing the original prefecture-level city powers, and plays a key role in national development, reform, and opening-up strategies. Zhoushan City, officially established in 1987, comprises the counties of Dinghai, Putuo, Daishan, and Shengsi, covering 1,390 islands, 1,440 km² of land, and 20,800 km² of inland sea. It serves as a typical example of administrative and functional integration.

We chose the Zhoushan Archipelago New Area for three main reasons: First, it is China’s first national new area dedicated to island development and the marine economy. Second, it is the only state-level new area that has provincial authority over economic and social management, which strengthens Zhoushan’s governance, even though its administrative level remains unchanged. Third, the creation of this new area allows Zhoushan to make the most of its strategic coastal location and port advantages, positioning it as a key player in national regional and marine development strategies.

#### Data sources and preliminary research.

As researchers, we are deeply involved in the study of administrative divisions and their impact on regional development, particularly in the context of China’s evolving urbanization and administrative reforms. Our primary interest in this topic stems from the broader challenges faced by policymakers in China when adjusting administrative levels and integrating functional zones. Leveraging the policy research project commissioned by the relevant government departments, we conducted an extensive field study in the Zhoushan Islands from June to August 2023. The primary focus of this research was on island new area planning, marine resources development, and local grassroots governance. During the fieldwork, we engaged in multiple work conferences organized by government departments, where they interacted with local officials and experts. Additionally, in-depth interviews were held with community leaders and representatives from local enterprises, providing valuable first-hand data for qualitative analysis.

We also gathered comprehensive socio-economic statistical data to support the analysis. This data was sourced from official publications such as the Zhoushan Statistical Yearbook (2009-2023), Ningbo Statistical Yearbook (2009-2023), Zhejiang Statistical Yearbook (2009-2023), and China Urban Statistical Yearbook (2009-2023). These sources provided detailed insights into the region’s demographic, economic, and industrial trends. For spatial information, data from the Resource and Environmental Science Data Center of the Chinese Academy of Sciences (http://www.resdc.cn) was used, with all spatial data preprocessed using ArcGIS software to ensure accuracy and consistency in the analysis.

This combination of field research and robust data collection laid the foundation for the development of the PLP model, helping to provide a comprehensive understanding of the factors influencing the PLP of the Zhoushan Archipelago New Area.

## Results

### The PLP change trend of the Zhoushan Archipelago New Area

The development plan for the Zhoushan Archipelago New Area was proposed in 2011, and in 2013, it was officially granted provincial-level social and economic management authority, elevating its administrative status. Using the PLP concept and calculation model, we compare the PLP of Zhoushan City with Ningbo City from 2008 to 2022, using the nearest city as a reference.

**Fig 2:**
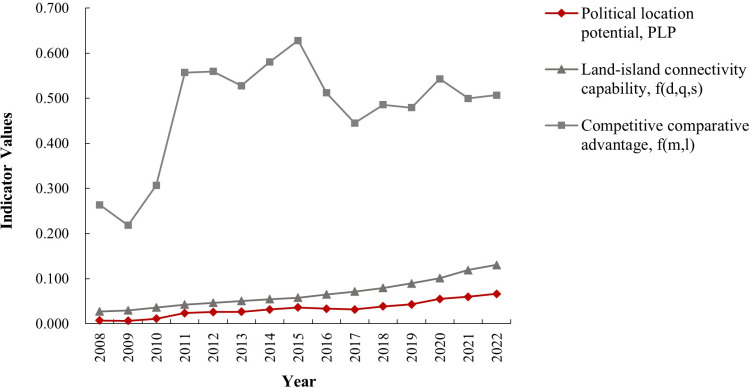
Political location potential and its sub variable changes in Zhoushan Archipelago New Area.

As shown in Fig 2, the PLP increased steadily from 2008 to 2022, but the rate of change varied at different stages. From 2010 to 2014, following the construction of the Zhoushan Cross-Sea Bridge and the establishment of the Zhoushan Archipelago New Area, the PLP grew from 0.011 to 0.032, with an average annual growth rate of 46.3%. The granting of provincial economic and social management authority in 2013 had a lagging effect, with noticeable changes occurring later. From 2014 to 2022, the average annual growth rate of the PLP was 5.51% in the first period and 17.8% in the second, suggesting that the policy’s effect became more prominent over time, significantly improving the comparative advantage of the administrative district.

Table 2 presents the relative potential energy of Zhoushan City every 5 years and before and after key policy changes from 2008 to 2022. Before and after Zhoushan was granted provincial-level economic and social management authority, the relative potential energy was 1.218, indicating a slight increase in PLP and a modest improvement in its regional comparative advantage. The relative potential energy was highest in the first stage, with the third stage outperforming the second, showing a shift from slow to rapid acceleration of Zhoushan’s locational comparative advantage due to the lagging policy effect.

**Table 2:** Relative potential energy across different time periods.

Time period (Year Range)	Relative potential energy (ΔY)	Time period (Year Range)	Relative potential energy (ΔY)
**2013–2008**	3.717	**2010–2008**	1.547
**2018–2013**	1.449	**2012–2010**	2.342
**2022–2013**	2.483	**2014–2012**	1.218
**2022–2008**	9.228	**2012–2010**	2.853

Further analysis of the relative potential energy before and after two key events—the opening of the Zhoushan Cross-Sea Bridge in 2009 and the granting of provincial-level authority in 2013—shows that in both stages, relative potential energy exceeded 1, aligning with the strategic goals of those periods. The primary objective of establishing the national-level archipelago new area, focusing on marine economy, was to highlight Zhoushan’s strategic location along China’s eastern coast and create a land-port link for economic resource circulation. The construction of the Cross-Sea Bridge played a crucial role in transforming Zhoushan into a national-level archipelago new area, enhancing its integration into the Yangtze River Delta as a maritime transportation hub, and driving regional economic growth.

### The direct effect of competitive comparative advantage

**Fig 3:**
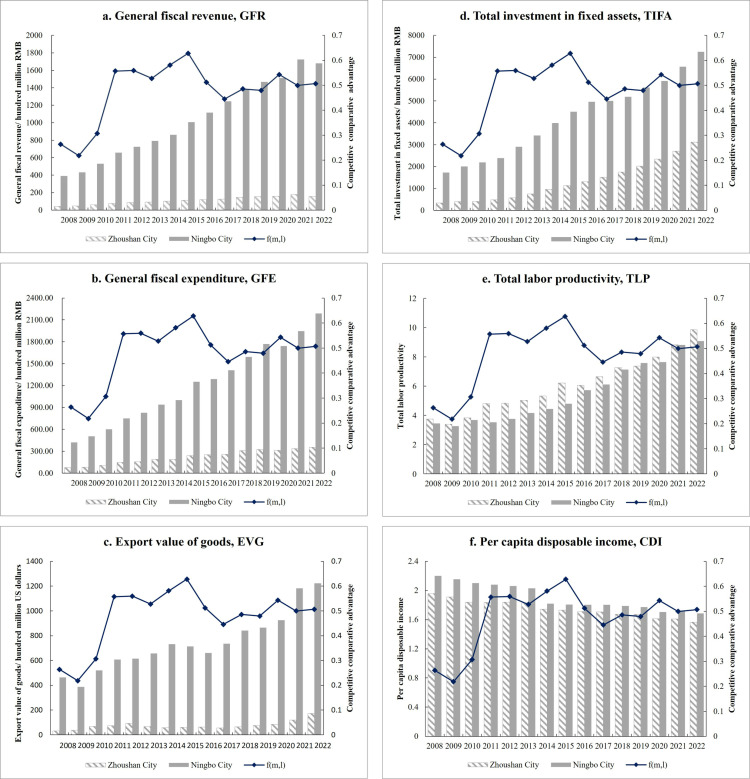
Competitive comparative advantage and sub-variable changes in Zhoushan City and Ningbo City. (a) **General fiscal revenue;** (b) **General fiscal expenditure;** (c) **Export value of goods;** (d) **Total investment in fixed assets;** (e) **Total labor productivity;** (f) **Per capita disposable income.**

The competitive comparative advantage value increased from 0.307 in 2010 to 0.581 in 2014, a growth rate of 89.3%, with the 2022 value reaching 0.507, nearly double that of 2008 (Fig 3). This indicates significant improvements in Zhoushan’s marine economy, driven by human resources and financial investments, which have a direct impact on the PLP by enhancing its competitive comparative advantage and strengthening its economic development capacity. With ongoing national policy support, Zhoushan has increased investment in fixed assets, fiscal spending, and human resources, while actively developing Zhoushan Port, promoting foreign trade, and fostering local economic strength. Fiscal reforms have helped Zhoushan achieve a balance between finance and economics, enhancing its competitive position relative to Ningbo City.

From 2008 to 2022, total labor productivity in Zhoushan increased from 3.754 to 9.854, and total fixed assets investment grew from 339.43 million yuan to 3.12 billion yuan, with growth rates of 39.04% and 132.19% between 2010 and 2014, respectively. These improvements are directly linked to the policy adjustments made after the establishment of the New Area, which have significantly boosted the city’s comparative advantage. As resource control continues to improve, Zhoushan’s competitive edge is expected to become even more prominent in the future.

Before the establishment of the Zhoushan Archipelago New Area, the city had a weak economic foundation and limited fiscal capacity. However, since 2013, national policies such as the rural revitalization strategy and the promotion of island prosperity have supported Zhoushan’s economic reforms. The city has increased fiscal investment, adjusted its structure, reduced enterprise burdens through tax cuts, and activated economic development. Fiscal revenue grew from 4.315 billion yuan in 2008 to 15.615 billion yuan in 2015, while fiscal expenditure rose from 7.678 billion yuan in 2008 to 35.427 billion yuan in 2022—almost a fivefold increase.

Notably, after reaching the 10-billion-yuan revenue mark in 2015, Zhoushan quickly surpassed 15 billion yuan in just four years, highlighting the substantial external investment attracted by its new administrative status. As China’s first national strategic new area focused on marine economy, Zhoushan capitalized on its foreign trade and maritime advantages. The city’s export value of goods rose from 3.29 billion USD in 2008 to 5.78 billion USD in 2014, and exceeded 10 billion USD by 2021, showing rapid growth and steady progress.

In 2024, the Zhejiang Provincial Institutional Reform Plan, approved by the Central Committee and the State Council, established the Zhejiang Provincial Department of Marine Economic Development. This institutional shift, along with the creation of Zhoushan’s Marine Economic Development Bureau, enhances coordination in marine planning, fisheries, and law enforcement, better positioning the city for high-quality marine economic development.

### The indirect effect of land-island connectivity capability

The establishment of the National Archipelago New Area has not only strengthened Zhoushan City’s administrative management authority but also created more opportunities for cooperation with neighboring cities, indirectly impacting the PLP by fostering regional synergies and enhancing collective development. The land-island connectivity capacity increased from 0.027 in 2008 to 0.131 in 2022, with the most significant growth occurring between 2009 and 2014, closely tied to the construction of cross-sea bridges and the establishment of the national-level new area.

Transportation infrastructure is key to opening up external connections for islands. The Zhoushan Cross-Sea Bridge, which opened in late 2009, significantly improved connectivity. We calculated the transportation accessibility between Zhoushan and Ningbo City, using the distance and shortest travel time from Ningbo’s South Bus Station to Yadanshan Pier in Zhoushan. Before the bridge, the waterway travel time between the cities was 1 hour and 50 minutes. After 2009, travel time was reduced to approximately 1 hour, and the journey from Zhoushan to Shanghai via the Hangzhou Bay Cross-Sea Bridge was shortened to 3 hours. These infrastructure developments, combined with the establishment of the National Archipelago New Area in 2011, marked a “three-level leap” for Zhoushan—from the island era to the bridge era, and then to the functional zone era. Economic indicators in Zhoushan align with the increased traffic flow through the Zhoushan Cross-Sea Bridge. Following the city’s granting of provincial-level management authority in 2013, economic strength increased by 19% from 2012 to 2014. Enhanced transportation has not only facilitated internal competition and cooperation but also supported external exchanges, bolstering Zhoushan’s geographical advantages.

Zhoushan’s permanent population has steadily grown since 2008, with the most significant increase occurring between 2008 and 2014. This period coincided with the opening of the Zhoushan Cross-Sea Bridge and the improvement of land-island connectivity, boosting the economy and population carrying capacity. After being granted provincial-level management authority in 2013, Zhoushan’s economic output, urban influence, and investment grew rapidly, creating a strong job market that attracted both returning residents and new migrants. These population shifts highlight the profound impact of the ADAs on the PLP, improving the district’s population attractiveness and competitiveness. Additionally, the ADA’s effects are often delayed, with significant changes becoming more apparent over time.

## Discussion

### Robustness testing of the model

To address the issue of spurious regression, we performed an ADF unit root test on the dependent variable and all independent variables. The results, shown in Table 3, reveal that the ADF statistics for the six variables—GFR, GFE, TIFA, TLP, EVG, and CDI—are all statistically significant at the 1% level. This indicates that these sub-variables became stationary after undergoing a second-order difference, which helps mitigate potential endogenetic issues.

**Table 3:** Results of Augmented Dickey-Fuller test.

Variables	ADF statistics	P-value	Lag Length	Conclusion
**General fiscal revenue (GFR)**	‒2.175	0.504	2	Non-Stationary
**General fiscal expenditure (GFE)**	‒3.34	0.001	1	Stationary
**Total investment in fixed assets (TIFA)**	‒3.205	0.083	2	Stationary
**Total labor productivity (TLP)**	‒3.368	0.056	2	Stationary
**Export value of goods (EVG)**	‒3.379	0.054	2	Stationary
**Per capita disposable income (CDI)**	‒4.869	0.001	1	Stationary

**Fig 4:**
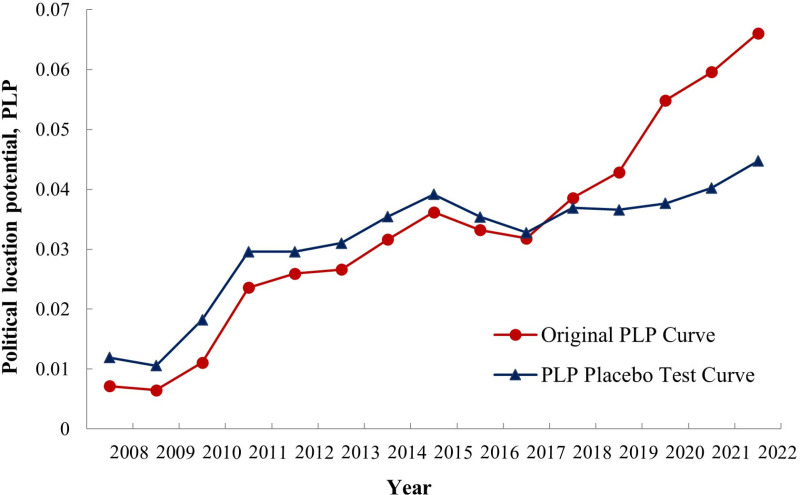
Changes in original PLP and PLP after replacing variables with “TIFA-EDE”.

To validate the robustness of our findings, we conducted a placebo test to ensure that the observed “policy effect” was not influenced by non-policy factors. In this test, the original independent variable, TIFA, was replaced with the education expenditure index (EDE), and the changes in PLP were recalculated (Fig 4). The results showed a consistent upward trend in PLP before and after the replacement, reinforcing the conclusion that the PLP model accurately captures the genuine advantages Zhoushan City gained after the establishment of the Zhoushan Archipelago New Area, free from external biases.

### Comparison of methods and results

As previously mentioned, the establishment of the Zhoushan Archipelago New Area granted Zhoushan City provincial-level social and economic management authority. Through quantitative analysis using the PLP model, we found that the increase in administrative authority enhances control over marine economic resources and administrative resource allocation, thereby boosting the island’s overall competitiveness. This adjustment aligns with the concept of ADAs, similar to Chongqing’s elevation to a municipality in 1997. Scholars have shown that since 1997, Chongqing’s PLP has steadily risen, demonstrating a long-term cointegration with economic performance indicators such as per capita GDP [5, 6]. The application of similar ADAs in various regions further supports these findings, confirming that adjustments in administrative levels can drive urban development in China [41].

It’s important to note that ADAs in China take various forms, including abolishing counties to establish districts or cities, adjusting municipal districts, and merging townships. Scholars have assessed the effects of these changes using methods like Difference-in-Differences, Synthetic Control, and Spatial Exploratory Analysis. These studies highlight the importance of administrative districts as critical policy tools within China’s governance system [42–44]. From the perspective of the PLP concept, these adjustments involve expanding the administrative scope of municipalities, thereby increasing control over land, human resources, administrative finances, and fixed asset investments, which in turn enhances the horizontal PLP. For counties or townships that are dissolved or downsized, their loss of administrative and financial authority reflects a shift in the vertical dimension of the PLP. Whether these adjustments lead to positive outcomes can also be quantitatively assessed using the PLP model. The advantage of using the PLP model lies in its ability to provide a comprehensive, data-driven framework to assess the impacts of these administrative adjustments. By quantifying both the horizontal and vertical changes in administrative authority and economic resources, the PLP model allows for a precise evaluation of how such changes affect local development.

Additionally, China’s “administrative district economy” phenomenon—resulting from the mismatch between administrative systems and economic reforms—causes regional economic fragmentation. The PLP model of integrating administrative and functional zones effectively mitigates this issue. Wang and Feng identified a coupling effect between PLP location and the administrative district economy, showing that administrative districts impose rigid constraints on regional integration [27]. The crux of the administrative district economy lies in the interaction between government control over administrative resources and the allocation of production factors. Starting from the institutional space, key variables in this dynamic are the control of island administrative resources and the allocation of marine economic resources. By analyzing the outcomes of this PLP model, we gain insights into how these factors collectively advance the island’s political standing. Moreover, integrating administrative and functional zones can help alleviate the negative effects of the administrative district economy.

### Recommendations for the integrated development of administrative and functional areas in Zhoushan

In China, functional areas are often referred to as “quasi-administrative districts” because they are not part of the formal administrative district system. These areas are established based on their administrative functions and the similarity of economic elements within them. To promote the integrated development of functional and administrative zones, China can explore several approaches, such as administrative oversight, merging functional and administrative zones, or transforming functional areas into formal administrative districts. In the long run, creating administrative districts represents the advanced stage of integrating functional zones, reflecting a region’s overall development potential. This integration is a distinct feature of the ADAs, and local governments should focus on leveraging its benefits to optimize regional structures, enhance resource allocation, elevate the administrative status of these areas, and boost their competitiveness in regional development.

The Zhoushan Archipelago New Area, China’s first national-level archipelago new area, includes five secondary economic functional zones: Marine Industry Cluster, New City Cluster, Putuo Mountain-Zhujiajian, Jintang, and Liuheng. These zones are under the jurisdiction of Zhoushan City. The zones are all county-managed, but at the township level, except for the Marine Industry Cluster, other zones integrate townships into the management committee’s office. This model weakens some township functions and hampers the core functions of the zones, resulting in low administrative efficiency. The lack of proper administrative division adjustments at the township level leads to coordination difficulties and redundant public administration processes. Therefore, optimizing administrative divisions and reforming the management system in Zhoushan City is crucial for promoting integrated development and achieving mutual benefits, as outlined in the three progressive administrative zoning adjustment pathways specifically tailored to the characteristics of the Zhoushan Archipelago New Area.

Firstly, adjusting mature functional areas to administrative districts. The New City Cluster Zone, which houses Zhoushan’s municipal government, has a land area of 88.6 km², a population of 117,000, and a fixed asset investment of 10.36 billion yuan, ranking first in both population agglomeration and economic strength. This area could be expanded by incorporating adjacent townships with similar characteristics, transitioning the zone from a functional area to an administrative district. The effectiveness of this adjustment can be evaluated using the PLP model, and if positive, it can be implemented in practice.

Secondly, optimizing township setting standards. Due to its archipelago nature, Zhoushan City has both land and island townships with different spatial distributions. For land-based towns, the criteria for setting up towns should be adjusted based on socio-economic factors like population density and economic output. Merging smaller towns to form larger administrative units can improve resource allocation and administrative efficiency. For island towns, spatial adjustments are more complex, so merging islands into community and village units, enhancing village autonomy, and integrating governance through digital grids will reduce costs and improve island development.

Thirdly, shifting functional area custodianship to municipal management. If adjusting administrative divisions is not feasible, Zhoushan Archipelago New Area could consider having the municipal government directly manage its five functional zones [45]. This would enable the municipal government to better coordinate industrial planning, construction, and financial support, aligning with the provincial-level social and economic authority granted to the new area.

### Limitations and future research

As an exploratory study, we present a novel perspective and methodology to quantitatively assess the impact of the ADAs from the new lens of the PLP. To empirically validate our approach, we selected the Zhoushan Archipelago New Area as a case study of the integration of administrative and functional areas. Our findings suggest that the PLP model offers a more comprehensive understanding of how the enhancement of administrative authority influences the socio-economic development of islands. However, there are certain limitations in both the mechanism analysis and model construction that warrant further consideration. The PLP model has limitations in fully accounting for inter-district cooperation, administrative costs, and the unique characteristics of different ADAs, which affect its accuracy and applicability, necessitating further refinement and the inclusion of additional variables for more precise assessments.

Firstly, the PLP model does not fully account for the cooperative relationships between administrative districts. In the context of China’s urban agglomeration development, the Zhoushan Archipelago New Area has fostered an integrated development trend with the neighboring city of Ningbo. Notably, the construction timelines of two cross-sea bridges overlap with the establishment of the national new area. The theory of regional competition and cooperation posits that such collaboration between administrative districts could create synergies that promote shared growth. While we attempted to capture this dynamic through the land-island connectivity level, future research should identify more precise variables to quantify the degree of cooperation or competition between administrative districts.

Secondly, the cost of local administration is a key factor that should be incorporated into the PLP model. The development model of administrative districts—whether it involves integrating administrative and functional areas or the independent development of functional areas—incurs different administrative costs, which in turn indirectly affect the magnitude of the PLP. Unfortunately, at present, comprehensive data on administrative costs in China is scarce. While we attempted to approximate these costs using general fiscal expenditure and other relevant indicators, it is important to also include local transfer payments and other data to better reflect fiscal decentralization and administrative costs.

Lastly, China’s ADAs are diverse, which means that the formula for measuring administrative district power must be adjusted to account for the unique characteristics of each administrative district and the specific type of ADA involved. For instance, while the Zhoushan Archipelago New Area emphasizes marine economy and administrative resource control, a resource-rich city focused on minerals might require additional indicators such as energy or mineral resource utilization efficiency. It is essential to acknowledge that any model will have limitations and cannot fully capture every internal mechanism. However, through continuous refinement and analysis, we can deepen our understanding of the PLP, offering new insights and reference points for future research on the impact of ADAs.

## Conclusion

Based on the Cobb-Douglas production function model, we have refined the original concept of the PLP by incorporating the unique characteristics of islands, resulting in the development of a quantitative measurement model for PLP. To validate this model, we applied it to the case of Zhoushan City, designated as China’s first and only national-level archipelago new area, and examined its PLP changes from 2008 to 2022. The findings of this study are as follows:

The PLP reflects the comparative advantage and ranking of an administrative district within a country or region. In island research, it can be measured across two dimensions: land-island connectivity capability and competitive comparative advantage. These dimensions encompass a range of direct and indirect variables, including administrative resource allocation, marine economic resource control, land-island transportation accessibility, and political area development scale.

The ADAs influence the PLP of an island through both horizontal changes in administrative management scale and vertical changes in administrative hierarchy. However, this impact typically exhibits a lag effect, meaning that the PLP of an island does not experience rapid improvements or declines in a short period. After a period of adaptation and accumulation, ADAs will ultimately have a positive impact on the PLP trend.

From 2008 to 2022, the overall PLP of Zhoushan City steadily increased, with specific years showing a relative potential energy greater than 1. Notable events, such as the opening of the cross-sea bridge in 2009, the establishment of the national archipelago new area in 2011, and Zhoushan’s granting of provincial-level social and economic management authority in 2013, were key milestones in this trend. The average annual rate of PLP change first increased, then decreased, with a decelerating pace, indicating that the establishment of the national-level archipelago new area significantly enhanced Zhoushan’s regional comparative advantage. However, the rate of improvement gradually slowed as the region reached a phase of more stable optimization.

Optimizing administrative divisions and reforming the management system in Zhoushan City is crucial for promoting integrated development and achieving mutual benefits. To this end, we propose three progressive administrative zoning adjustment pathways tailored specifically to the characteristics of the Zhoushan Archipelago New Area. These pathways aim to further integrate administrative districts and functional areas, enhancing Zhoushan’s governance and regional competitiveness.

The PLP model proposed in this study serves as a benchmark that can be adapted to assess the impacts of various ADA plans, such as the abolition of counties, merging administrative districts, the creation of cities by dissolving counties, and changes in administrative subordination relationships, on local development. This model offers valuable insights for governments at all levels, urging them to prioritize the positive effects of ADAs. By optimizing and adjusting the structure of administrative districts and promoting the effective integration of administrative districts and functional areas, they can enhance the competitiveness and development capabilities of administrative regions within broader regional development.

## Supporting information

S1 File Raw Data for PLP Calculation. (XLSX)
